# Splitting unramified Brauer classes by abelian torsors and the period-index problem

**DOI:** 10.1007/s00208-025-03161-2

**Published:** 2025-04-26

**Authors:** Daniel Huybrechts, Dominique Mattei

**Affiliations:** Mathematisches Institut & Hausdorff Center for Mathematics, Universität Bonn, Endenicher Allee 60, 53115 Bonn, Germany

## Abstract

We use twisted relative Picard varieties to split Brauer classes on projective varieties over algebraically closed fields by torsors for a fixed abelian scheme independent of the Brauer class. The construction is also used to prove that the index of an unramified Brauer class divides a fixed power of its period.

## Introduction

We begin by stating the two main results of the paper. For motivation and further comments on the history we refer to Sects. 1.3 & 1.4.

1.1. Our first theorem shows that Brauer classes on a projective variety can be split after pull-back to torsors for abelian varieties, its proof will be explained in Sect. 3 and concluded in Sect. 3.3.

### Theorem 1.1

Let *X* be an integral projective variety over an algebraically closed field *k* and let *K*(*X*) be its function field. Then there exists an abelian variety *A* over *K*(*X*) such that for every Brauer class $\alpha \in {\textrm{Br}}(X)$ one finds an *A*-torsor $B_\alpha$ that splits $\alpha$.

In other words, for every $\alpha \in {\textrm{Br}}(X)$ the compositionmaps $\alpha$ to the trivial Brauer class on $B_\alpha$. Thus, the theorem shows how to split unramified Brauer classes over the function field *K*(*X*) by passing to torsors for a fixed abelian variety.

We emphasise that our construction produces an abelian variety *A* that is independent of the Brauer class $\alpha$, but the torsor $B_\alpha$ does depend on $\alpha$. However, as all the $B_\alpha$ are torsors for the same abelian variety *A*, their dimensions are independent of $\alpha$. Let us also point out that typically *A* and $B_\alpha$ are not unique and may even come in families.

1.2. The second main result concerns the period–index problem. Recall that the period of a central simple algebra *A* over a field *K* is by definition the order $\textrm{per}(A)=\textrm{per}(\alpha )=|\alpha |$ of its class $\alpha =[A]\in {\textrm{Br}}(K)$ in the Brauer group of *K*. Its index is defined as $\textrm{ind}(A)=\textrm{ind}(\alpha )=\dim (D)^{1/2}$, where *D* is the unique division algebra such that $A\simeq M_n(D)$ for some *n*. It is known classically that $\textrm{per}(D)\mid \textrm{ind}(\alpha )$ and that both invariants share the same prime factors, i.e. $\textrm{ind}(\alpha )\mid \textrm{per}(\alpha )^{e_\alpha }$ for some integer $e_\alpha$ which a priori depends on $\alpha$, cf. [7, (5.3)] or [23, Prop. 4.5.13].

Our second theorem shows that for unramified Brauer classes over the function field *K*(*X*) of a projective variety, i.e. for classes in the image of , the exponent $e_\alpha$ can be chosen uniformly. The proof is presented in Sect. 4.

### Theorem 1.2

Let *X* be an integral projective variety over an algebraically closed field. Then there exists an integer *e* such that for all unramified classes $\alpha \in {\textrm{Br}}(K(X))$ one has

$$\begin{aligned} \textrm{ind}(\alpha )\mid \textrm{per}(\alpha )^{e}. \end{aligned}$$

The exponent appearing in the theorem is $e=2g(C)$, where *g*(*C*) is the genus of a very ample complete intersection curve on *X*.

This uniform bound for the index is a consequence of the same geometric construction of the torsors used to prove Theorem 1.1. For abelian varieties, a divisibility with the same exponent had been remarked before by Bhatt [30, App.] and Clark [12, 13].

Section 2 treats the case of elliptic K3 surfaces, with or without a section. It serves as a warm-up to the general case, but it also proves more specific results in this situation.

### Splitting Brauer classes

Splitting Brauer classes by passing to higher-dimensional varieties is nothing unusual. For example, if  is a Brauer–Severi variety representing the Brauer class $\alpha \in {\textrm{Br}}(X)$ then $\pi ^*(\alpha )$ is the trivial class in ${\textrm{Br}}(P_\alpha )$. However, the dimension of $P_\alpha$ depends on $\alpha$ and cannot be uniformly bounded.

Since for any subvariety $Y\subset P_\alpha$ the projection  also splits $\alpha$, i.e. $\pi _Y^*(\alpha )$ is the trivial class in ${\textrm{Br}}(Y)$, one can use hyperplane sections of the projective variety $P_\alpha$ to produce a projective variety  of relative dimension one with irreducible generic fibre that splits a given Brauer class $\alpha$. So, the dimension of the variety to split $\alpha$ can be bounded. However, with this construction the genus of the generic fibre will be unbounded for varying $\alpha$.

In fact, it is known that if a smooth projective curve *C* of genus *g* over a field *K* splits a Brauer class $\alpha \in {\textrm{Br}}(K)$, then the order of $\alpha$ divides $2g-2$, cf. [2, Intr.] or [24, Proof of Thm. 1.0.2 ]. Thus, when its genus is at least two, it is bounded from below by $(1/2)\cdot |\alpha |$.

Whether every Brauer class $\alpha \in {\textrm{Br}}(X)$, or more generally $\alpha \in {\textrm{Br}}(K)$, is split by a genus one curve $C_\alpha$ is a rather recent question. In this generality, it was first raised by Clark and Saltman, cf. [12, 41], but with earlier results for local fields by Roquette [40]. The question is wide open but it was answered positively for low index, see [4, 5, 19]. In Sect. 2.1 we settle the case of Brauer classes on elliptic K3 surfaces with a section.

Note that in light of our result one could ask the even stronger question whether every class $\alpha \in {\textrm{Br}}(X)$ is split by a torsor for some fixed elliptic curve *E* over *K*(*X*). However, doubts already about the weaker version have been raised by de Jong and Ho [19] and it seems likely that Theorem 1.1 is the best result in this direction one can hope for in general. Note however that Antieau and Auel [2, Thm. C] show that every cyclic Brauer class over a field containing an algebraically closed field is split by a torsor for an elliptic curve.

Theorem 1.1 can be seen as an improvement of a result by Ho and Lieblich [24], it essentially answers their Question 6.0.1 for unramified classes, and by Antieau and Auel [2, Thm. 3.18]. More concretely, in [24] it is shown that for a fixed field *K* there exists for every class $\alpha \in {\textrm{Br}}(K)$ of index $\not \equiv 2\,(4)$ a smooth projective curve $C_\alpha$ such that $\alpha$ is split by its Albanese $\textrm{Alb}(C_\alpha )=\textrm{Pic}^1(C_\alpha )$, a torsor for the abelian variety $\textrm{Pic}^0(C_\alpha )$. If the index is $\equiv 2(4)$, then $\alpha$ is split by $C'\times \textrm{Pic}^1(C_\alpha )$, where $C'$ is an additional curve of genus one. In both cases, the curve $C_\alpha$ itself is obtained by taking complete intersections in a Brauer–Severi variety representing $\alpha$ as described above. In particular, its genus and hence the dimension of $\textrm{Pic}^1(C_\alpha )$ depend on the index of $\alpha$ and cannot be bounded. As was explained to us by Auel, splitting by torsors for abelian varieties can also be achieved by using the Merkurjev–Suslin theorem [37], cf. [23, Ch. 8], which shows that ${\textrm{Br}}(K(X))$ is generated by cyclic classes. Combined with a result by Matzri [36] bounding the symbol length of Brauer classes over function fields, the dimension of the abelian variety can be again bounded in terms of the period of the Brauer class. The argument in [2, §3] uses instead a result of Raynaud about realizing finite abelian group schemes as subschemes of abelian schemes.

In [24] one finds further questions about splitting Brauer classes. Torsors for elliptic curves and abelian varieties seem geometrically the most interesting cases, but one could try to use K3 surfaces or Calabi–Yau varieties just as well. We have nothing to say about those.

### Period–index problem

The period–index problem, asking for an exponent $e_\alpha$ that is independent of $\alpha$, has a long history. For number fields, it has been proved by Albert, Brauer, Hasse, and Noether [1, 8] that $\textrm{per}(\alpha )=\textrm{ind}(\alpha )$, i.e. $e_\alpha =1$, and the same holds for function fields of transcendence degree one over finite fields.

In the geometric situation, where *K* is the function field of a curve over an algebraically closed field, the question is vacuous by Tsen’s theorem. For surfaces, de Jong [18] proved $\textrm{per}(\alpha )=\textrm{ind}(\alpha )$, so again $e_\alpha =1$, for Brauer classes $\alpha$ with $\textrm{per}(\alpha )$ prime to the characteristic of the ground field, so in particular for all classes in characteristic zero. Later, de Jong and Starr [20] and Lieblich [35] extended the result to all classes. Already in [15], Colliot-Thélène proposed the bound $e_\alpha =\dim (X)-1$ in arbitrary dimensions, see also [16, §2.4], and Kahn suggested $e_\alpha =\lceil {\dim (X)/2}\rceil$ in [29].

In [21, Conj. 1.2], de Jong and Perry formulated a weaker a conjecture that asks for a bound $e=e_\alpha$ that only depends on the variety *X* and not on the (unramified) Brauer class $\alpha$. Moreover, they proved the existence of such a uniform bound under the assumption that the Lefschetz standard conjecture holds true in degree two. Theorem 1.2 proves their conjecture unconditionally.

It is known that techniques developed by de Jong and Starr [20, 44] allow one to deduce the conjecture $e_\alpha =\dim (X)-1$ for all Brauer classes from the case of unramified ones. Unfortunately, as our exponent depends on the geometry of *X* and not only on its dimension, this argument does not work which limits our approach to the case of unramified Brauer classes.

The proof of Theorem 1.2 relies on the construction of a module $V_\alpha$ of bounded dimension over the Azumaya algebra *A* representing $\alpha$. This module $V_\alpha$ is eventually constructed as a space of theta functions on the twisted Picard variety $B_\alpha$, see (4.1) in Sect. 4.1.

After the first version of our paper appeared on the arxiv, an alternative argument for Theorem 1.2 was communicated to us by M. Lieblich and also by B. Antieau (coming out of an independent discussion with A. Auel). Colliot-Thélène informed us that also D. Krashen, has independently found a proof for the existence of a uniform bound.

The geometric setup is the same. The tensor product  is used to define a morphism  from a twisted Picard variety to an untwisted one. In our approach, the module $V_\alpha$ alluded to above is constructed as the space of global sections of the pull-back of the theta divisor twisted by the universal Poincaré bundle. The argument simplifies somewhat, if instead restriction to the finite kernel of the morphism is used.

### Twisted Picard varieties

The key idea to approach Theorem 1.1 is very simple. We use a dominating universal family of smooth (complete intersection) curves  in *X* parametrised by a quasi-projective variety *T* and consider the twisted relative Picard variety $\textrm{Pic}_\alpha ^d(\mathcal {C}/T)$. These twisted Picard varieties are special cases of moduli spaces of twisted sheaves constructed by Lieblich [33] and Yoshioka [45], but can also be viewed as instances of Simpson’s moduli space of $\Lambda$-modules [43], see also [25] for the case of positive characteristic. They have recently been studied for K3 surfaces in [28] and we use the notation introduced there. For an appropriate choice of $d\in \mathbb {Q}$, the projection  is a torsor for the abelian scheme  and in the ideal situation $\textrm{Pic}_\alpha ^d(\mathcal {C}/T)$, as a moduli space of twisted sheaves on *X*, is fine. Since the universal family over $\mathcal {C}\times _T\textrm{Pic}_\alpha ^d(\mathcal {C}/T)$ is twisted with respect to the pull-back of $\alpha$ and it is of rank one, the pull-back of $\alpha$ is split.

The final step is to shrink  to  such that the projection  is birational. Then the projection  provides the torsor claimed in Theorem 1.2.

Let us be a bit more explicit about the moduli space that we wish to exploit. As a first step, we pick an Azumaya algebra $\mathcal{A}$ on *X* that represents the Brauer class $\alpha$. Then $\textrm{Pic}^d_\alpha (\mathcal {C}/T)$ is constructed as an open subset of a moduli space of modules over $\mathcal{A}$ supported on curves $\mathcal {C}_t\subset X$, $t\in T$, cf. [43]. The universal family is then a sheaf $\mathcal{P}$ on $\mathcal {C}\times _T\textrm{Pic}_\alpha ^d(\mathcal {C}/T)$ which is a module over the pull-back $\mathcal{A}'$ of $\mathcal{A}$ under the projection to *X*. By definition of the twisted Picard variety as a moduli space of certain sheaves, the rank of $\mathcal{P}$ is $d(\mathcal{A}):=\sqrt{\textrm{rk}(\mathcal{A})}$. This implies that the pull-back of $\alpha$ is trivial, for $\mathcal{A}'\simeq \mathcal {E}\hspace{-1.66656pt}\mathcal {n}\mathcal {d}(\mathcal{P})$ on a dense Zariski open subset.

There are various ways of dealing with moduli spaces in a twisted situation. Each of them involves an additional choice, e.g. of a Čech cocycle, an Azumaya algebra, a Brauer–Severi variety, or a $\mu _n$-gerbe, representing the fixed Brauer class. The moduli space is then constructed as a space of sheaves twisted with respect to the Čech cocycle, of modules over the Azumaya algebra, of sheaves on the Brauer–Severi variety, or of invertible sheaves of weight one on a $\mu _n$-gerbe. We decided to represent the Brauer class by an Azumaya algebra but every other choice would have worked just as well.

In the end the triviality of the pull-back $\alpha '$ of $\alpha$ follows from the existence of a locally free $\alpha '$-twisted sheaf of rank one or, in terms of Azumaya algebras, from the existence of a locally free $\mathcal{A}'$-module of rank $d(\mathcal{A})$, where $\mathcal{A}$ represents $\alpha$ and $\mathcal{A}'$ is its pull-back.

## Warmup: elliptic curves on K3 surfaces

Before presenting the general proof of Theorem 1.1, we discuss the special case of elliptic K3 surfaces. It is the first instance where universal sheaves are seen to split Brauer classes and it is instructive to study this case first before dealing with the general situation in the next section. As it seems more customary, we here work with twisted sheaves instead of modules over Azumaya algebras, but this does not effect the essence of the argument.

We will find that for elliptic surfaces with a section all Brauer classes are split by a genus one fibration (over a fixed elliptic curve) and so Clark’s original questions has an affirmative answer in this case, see Proposition 2.1. For elliptic K3 surfaces without a section, only Brauer classes of an order coprime to the minimal degree of a multi-section of the elliptic fibration can be split by the same method, see Proposition 2.3.

We will conclude this section by discussing families of (singular) K3 surfaces. Something can still be said but the result is less compelling, see Proposition 2.4.

In fact, the discussion in this section applies to elliptic surfaces, with and without a section, that are not necessarily K3 surfaces. However, for simplicity and as the main purpose of this section is only to illustrate the proof in the general setting, we stick to K3 surfaces.

### Elliptic K3 surfaces with a section

Consider an elliptic K3 surface  with a section and a class . By definition of the Tate–Šafarevič group , the class $\alpha$ corresponds to an elliptic K3 surface  (without a section) together with an isomorphism $\textrm{Pic}^0(S/\mathbb {P}^1)\simeq S_0$ relative over $\mathbb {P}^1$. Here, $\textrm{Pic}^0(S/\mathbb {P}^1)$ denotes the minimal smooth compactification of the Jacobian fibration of the family of smooth fibres of . Such a compactification is provided by the moduli space *M*(0, *f*, 0) of stable sheaves on *S* with Mukai vector (0, *f*, 0), where *f* is the class of a fibre, cf. [27, Ch. 11]. However, such a moduli space is not fine, i.e. there is no universal family on $S_0\times S$.

The obstruction for a universal family to exist is a class in ${\textrm{Br}}(S_0)$. In fact, this class is nothing but $\alpha$ itself under the well-known isomorphism , cf. [27, Prop. 11.5.6]. To be more precise, the obstruction class in ${\textrm{Br}}(S_0)$ is the class of the Čech cocycle that comes up naturally when we try to glue the local (étale or analytic) universal families, which always exist. This point of view also shows that a universal family $\mathcal{P}$ exists as a twisted sheaf $\mathcal{P}$ on $S\times S_0$, where the twist is with respect to the pull-back of the cocycle on $S_0=\textrm{Pic}^0(S/\mathbb {P}^1)$ representing the obstruction class $\alpha$. The construction was systematically studied by Cǎldǎraru [11].

By definition of the moduli space, the $(1\times \alpha )$-twisted sheaf $\mathcal{P}$ on $S\times S_0$ has support on the closed subscheme $S\times _{\mathbb {P}^1}S_0\subset S\times S_0$. Furthermore, there it is of rank one. Hence, on a non-empty Zariski open subset $V\subset S\times _{\mathbb {P}^1}S_0$, the twisted sheaf $\mathcal{P}$ is locally free of rank one which implies that $(1\times \alpha )|_V$ is a trivial Brauer class.

The generic fibre of the second projection  is a smooth curve of genus one. More precisely, it is the generic fibre $S_\zeta$ of  base changed to the generic point $\eta \in S_0$ which, of course, maps to $\zeta$ under the projection . We have proved the following result.

#### Proposition 2.1

Let  be an elliptic K3 surface with a section and let $\alpha \in {\textrm{Br}}(S_0)$. Then there exists a genus one curve $C_\alpha$ over its function field $K(S_0)$ such that the image of $\alpha$ under the composition  is trivial. $\square$

We can be more precise about the curve $C_\alpha$. By construction, it is the generic fibre of  which is the base change to $K(S_0)$ of the moduli space of line bundles on the generic fibre of  twisted with respect to the restriction of $\alpha$. But this moduli space is naturally a torsor for $\textrm{Pic}^0$ of the generic fibre of  where the action is given by tensor product.

Since  has a section, its relative $\textrm{Pic}^0$ is just  itself. Hence, all the genus one curves $C_\alpha$ in Proposition 2.1 are torsors for the same elliptic curve, namely the base change to $K(S_0)$ of the generic fibre of .

### Elliptic K3 surfaces without a section

Since in the above discussion, the K3 surface *S* could be seen as a certain moduli space $\textrm{Pic}^d_\alpha (S_0/\mathbb {P}^1)$ of twisted sheaves on the fibres of , cf. [28], one could try to extend the argument to elliptic K3 surfaces  without a section by simply considering $\textrm{Pic}^d_\alpha (S_0/\mathbb {P}^1)$. Strictly speaking, the notation $\textrm{Pic}^d_\alpha (S_0/\mathbb {P}^1)$ makes only sense when a lift of $\alpha$ to an element in the special Brauer group $\textrm{SBr}(S_0)$ has been chosen: Only then the degree *d* is well defined. The special Brauer group has been introduced by Grothendieck [9, Rem. 3.9] and [28, §2] contains a detailed discussion of this notion in the present context. The lift is only needed to index the various components of the moduli space of all semi-stable twisted sheaves of rank one on the fibres. For our purpose, $\textrm{Pic}^d_\alpha (S_0/\mathbb {P}^1)$ just denotes one of the components, which we have to choose suitably, but see also Sect. 3.2.

So the idea would be to use a universal family $\mathcal{P}$ on $\textrm{Pic}^d_\alpha (S_0/\mathbb {P}^1)\times _{\mathbb {P}^1}S_0$ to argue that the pull-back of $\alpha$ under the second projection has to be trivial. And indeed, by definition of $\textrm{Pic}_\alpha ^d(S_0/\mathbb {P}^1)$ as a moduli space of $\alpha$-twisted sheaves, $\mathcal{P}$ would be twisted with respect to $\alpha$ on the second factor.

However, there is no guarantee that such a universal family $\mathcal{P}$ exists as a $(1\times \alpha )$-twisted sheaf, i.e. that the moduli space $\textrm{Pic}^d_\alpha (S_0/\mathbb {P}^1)$ is a fine moduli space of twisted sheaves on $S_0$. In other words, $\mathcal{P}$ might only exist as a sheaf that is not only twisted by $\alpha$ on the second factor but also by some obstruction class on the first. If this is the case, the argument breaks down and we cannot conclude the triviality of the pull-back of $\alpha$.

However, something can be salvaged from this approach by exploiting a certain flexibility in the choice of *d* (after choosing a lift of $\alpha$ to a class in the special Brauer group). For this we make use of a well-known criterion for $\textrm{Pic}^d_\alpha (S_0/\mathbb {P}^1)=M_\alpha (0,f,d)$ to be a fine moduli space: It suffices to find an $\alpha$-twisted locally free sheaf *E* such that $\chi (F\otimes E^*)=1$ for $F\in \textrm{Pic}^d_\alpha (S_0/\mathbb {P}^1)$. See [26, Ch. 4.6] for the untwisted case, the proof in the twisted case is identical.

#### Lemma 2.2

Assume the order of $\alpha \in {\textrm{Br}}(S_0)$ is coprime to the generator *m* of $(\textrm{NS}(S_0).f)$. Then there exists a (twisted) degree $d\in \mathbb {Q}$ with $\textrm{Pic}^d_\alpha (S_0/\mathbb {P}^1)$ non-empty and an $\alpha$-twisted locally free sheaf *E* on *S* such that $\chi (F\otimes E^*)=1$ for every *F* parametrised by $\textrm{Pic}^d_\alpha (S_0/\mathbb {P}^1)$. In particular, the moduli space $\textrm{Pic}_\alpha ^d(S_0/\mathbb {P}^1)$ of $\alpha$-twisted sheaves on $S_0$ is fine.

#### Proof

We start with any $\alpha$-twisted line bundle *F* supported on a smooth fibre $S_{0t}$ of  that defines a point in some non-empty moduli space $\textrm{Pic}_\alpha ^d(S_0/\mathbb {P}^1)$. Let us also fix some $\alpha$-twisted locally free sheaf *E* on $S_0$ of rank $|\alpha |$. The existence of the latter is guaranteed by de Jong’s solution [18] of the period-index problem for algebraic surfaces and its generalisation to positive characteristic [20, 35].

We will now modify *E*, keeping it $\alpha$-twisted and locally free of rank $|\alpha |$, and *F*, possibly changing *d* in the process, such that eventually $\chi (F\otimes E^*)=1$.

Observe first that the kernel $F'$ of any surjection , $x\in S_{0t}$, defines again an $\alpha$-twisted line bundle on $S_{0t}$ which satisfies $\chi (F'\otimes E^*)=\chi (F\otimes E^*)-|\alpha |$. Second, we pick a curve $C\subset S_0$ with $(C.f)=m$^1^ and consider an elementary transformation $E'$ of *E* along *C*, i.e. $E'$ is the $\alpha$-twisted locally free sheaf given as the kernel of some surjection  onto some $\alpha$-twisted line bundle $\mathcal{L}$ on *C*. Then $\chi (F\otimes E'^*)=\chi (F\otimes E^*)-m$.

Since $|\alpha |$ and *m* are coprime, applying the procedure multiple times eventually provides us with *E* and *F* as claimed. $\square$

Note that in particular we reprove the assertion in the case that  has section, for then $(\textrm{NS}(S_0).f)=\mathbb {Z}$ and hence $m=1$. If there is no section, then the discussion eventually leads to the following generalisation of Proposition 2.1.

#### Proposition 2.3

Let  be an elliptic K3 surface and let $m\in \mathbb {Z}$ be such that $m\cdot \mathbb {Z}=(\textrm{NS}(S_0).f)$ for the fibre class *f*. Then one finds an elliptic curve *E* over $K(S_0)$ such that for every $\alpha \in {\textrm{Br}}(S_0)$ of order $|\alpha |$ coprime to *m* there exists a torsor $C_\alpha$ for *E* over $K(S_0)$ such that the image of $\alpha$ under  is trivial. $\square$

### Families of elliptic curves on arbitrary K3 surfaces

Every K3 surface admits a covering family of elliptic curves, cf. [27, Cor. 13.2.2]. Does this mean that Brauer classes on arbitrary K3 surfaces are split by curves of genus one? Unfortunately, our approach fails in this generality, but something can still be said.

For any K3 surface *S* there exists a one-dimensional smooth family of curves of genus one  over a smooth curve *T* with a dominant map . Following the same strategy as in the last two sections, one considers . As soon as $\textrm{Pic}_\alpha ^d(\mathcal {C}/T)$ is a fine moduli space, which can again be phrased as a numerical condition on $|\alpha |$ being coprime to a fixed integer *m*, there exists a universal family of $\alpha$-twisted sheaves on $\textrm{Pic}_\alpha ^d(\mathcal {C}/T)\times _T\mathcal {C}$. This, as before, implies that the pull-back of $\alpha$ is trivial. Now,is a family of curves of genus one, generically a torsor for .

The difference to the case of elliptic K3 surfaces, with or without a section, is that the projection  is typically not birational. In other words, there often exists more than one elliptic curve parametrised by *T* passing through a generic point in *S*. Thus, by this method, one only proves the following.

#### Proposition 2.4

Let *S* be a complex projective K3 surface. Then there exists an integer $m>0$, a finite extension $K'/K(S_0)$, and an elliptic curve *E* over $K'$ such that for every Brauer class $\alpha \in {\textrm{Br}}(S)$ of order $|\alpha |$ coprime to *m*, one finds a torsor $C_\alpha$ for *E* over $K'$ such that under  the image of $\alpha$ is trivial. $\square$

Note that any Brauer class splits under some finite extension of $K(S_0)$. So the interest of the last proposition stems from the fact that $K/K(S_0)$ is a fixed finite extension that works for all Brauer classes (of order coprime to *m*).

#### Remark 2.5

The techniques here would certainly also apply to other types of surfaces with a covering family of (singular) elliptic curves and would prove splitting of Brauer classes by genus one curves over a fixed finite extension of the function field. However, apart from elliptic surfaces, those are only abelian, K3 and Enriques surfaces and surfaces with trivial Brauer group. See also Remark 3.5.

### Period–index problem for elliptic K3 surfaces with a section

We now outline the proof of Theorem 1.2 in the case of elliptic K3 surfaces with a section, which might again serve as an illustration of the argument in the general situation. Here, we use the language of twisted sheaves while in the actual proof in Sect. 4 we employ sheaves over Azumaya algebras.

As in Sect. 2.1, we assume that  is an elliptic K3 surface with a section and that  is the elliptic K3 surface corresponding to a fixed class . By definition of the Tate–Šafarevič group, $\textrm{Pic}^0(S/\mathbb {P}^1)\simeq S_0$, but the Poincaré bundle $\mathcal{P}$ on the fibre product $S\times _{\mathbb {P}^1}S_0$ only exists as $(1\times \alpha )$-twisted invertible sheaf $\mathcal{P}$. Via the second projection , this fibre product is viewed (over a dense open subset of $S_0$) as a family of smooth curves of genus one (the smooth fibres of ) and $\mathcal{P}$ is a family $\mathcal{P}|_{S_{\pi (x)}}$, $x\in S_0$, of untwisted line bundles of degree zero on these fibres.

It is is known that the period $\textrm{per}(\alpha )=|\alpha |$ coincides with the minimal fibre degree of invertible sheaves on . The result is due to Ogg [38], Šafarevič [42], and Lichtenbaum [31, Thm. 1], see also [39] for a thorough discussion of the relevant obstruction. Alternatively, it can be deduced from global arguments involving the transcendental lattice of the K3 surfaces, cf. [27, Remark 11.5.9]. Thus, there exists a line bundle *M* on *S* such that $\deg (M|_{S_t})=\textrm{per}(\alpha )$. Hence, the restrictions of $\mathcal{P}\otimes ({\textrm{pr}_1^*M})$ to the fibres of $\textrm{pr}_2$ are invertible sheaves of degree $|\alpha |$ and, therefore, the direct image ${\textrm{pr}_{2*}}\mathcal{P}$ is a torsion free, $\alpha$-twisted sheaf of rank $\textrm{per}(\alpha )$. This immediately implies that $\textrm{ind}(\alpha |_{K(X)})=\textrm{per}(\alpha )$.

This proves the following special case of de Jong’s result [18] for elliptic K3 surfaces with a section.

#### Proposition 2.6

Assume  is an elliptic K3 surface with a section. Then

$$\begin{aligned} \textrm{per}(\alpha )=\textrm{ind}(\alpha ) \end{aligned}$$

for every class $\alpha \in {\textrm{Br}}(S_0)\subset {\textrm{Br}}(K(S_0))$. $\square$

## Twisted relative Picard varieties

Before entering the proof of Theorem 1.1, let us rephrase the result geometrically. The assertion is that there exists a variety  which generically is an abelian scheme over *X* such that for every Brauer class $\alpha \in {\textrm{Br}}(X)$ one finds a variety  generically a torsor for $\textbf{A}$ such thatmaps $\alpha$ to a trivial class on $\textbf{B}_\alpha$.

The passage from Theorem 1.1 to the more geometric version is obtained by viewing $B_\alpha$ as the generic fibre of $\textbf{B}_\alpha$ dominating *X*. In characteristic zero, we can assume that $\textbf{B}_\alpha$ is smooth (locally factorial would be enough) and projective. Indeed, then the restriction map  to the generic fibre is injective. Otherwise, if $\textbf{B}_\alpha$ cannot be resolved by a locally factorial variety, one might have to restrict to a Zariski open neighbourhood of the generic fibre $B_\alpha$, so that $\textbf{B}_\alpha$ would not necessarily be projective anymore.

After introducing the setup, Sect. 3.2 proves a version of Theorem 1.1, namely the existence of *A* and $B_\alpha$ not over the function field of *X*, but over some universal extension of it obtained by the family of complete intersection curves. Section 3.3 explains how the universal family of all complete intersection curve is cut down to a family that dominates *X* birationally.

### Setting: complete intersection curves

Let us set the stage for the proof of Theorem 1.1. We let *X* be an integral projective variety of dimension *n* over an algebraically closed field *k*. Passing to its normalisation, we may assume from the start that *X* itself is normal. In characteristic zero we can even assume that *X* is smooth, but we will not need this.

Next, we fix a very ample linear system $|h| \simeq \mathbb {P}^N_k$ on *X* and produce a family of complete intersection curvesof smooth curves $\mathcal {C}_t\subset X$ parametrised by an open dense subset *U* of the Grassmannian ${{\mathbb {G}}}(n-2,|h|)=\textrm{Gr}(n-1,H^0(X,\mathcal{O}(h)))$. Later it will be convenient to assume that all curves parametrised by *U* are contained in the smooth part of *X*, for which we will need the normality of *X*. Indeed, if *X* is normal, the singular set of *X* has codimension at least two, so that the generic one-dimensional complete intersection of ample hypersurfaces will avoid it. Also note that we may assume $\dim (X)\ge 2$, so that we really can talk about a family of curves contained in *X*. Indeed, the Brauer group of a smooth projective curve over an algebraically closed field is trivial.

The relative Picard scheme  of the family will be central for our discussion and eventually leads to the abelian variety *A*.

### Twisted relative Picard variety

For any class $\alpha \in \textrm{SBr}(X)$ in the special Brauer group $\textrm{SBr}(X)$, i.e. for any $\mu _n$-gerbe in $H^2(X,\mu _n)$ lifting a Brauer class in $H^2(X,{{\mathbb {G}}}_m)$, and any $d\in \mathbb {Q}$, we can consider the relative twisted Picard variety, cf. [28],which, if not empty, is a torsor for the Picard scheme . Its pull-back under  provides us with a torsorfor the abelian scheme . Altogether, we have the commutative diagram3.1Let us spell this out a bit more. First pick an Azumaya algebra $\mathcal{A}$ on *X* representing the class $\alpha \in \textrm{SBr}(X)$. Then $\textrm{Pic}^d_\alpha (\mathcal {C}/U)$ is an open subset of the moduli space of stable $\mathcal{A}$-modules *E* on *X* supported on complete intersection curves $C=H_1\cap \ldots \cap H_{n-1}\subset X$ with $H_i\in |h|$.

Moduli spaces of this type are a special case of Simpson’s moduli spaces of $\Lambda$-modules, cf. [43]. Alternative construction via gerbes or Brauer–Severi varieties are due to Lieblich [33] and Yoshioka [45]. There are some subtleties in comparing the stability conditions, see e.g. [32, §4.1.4], but for locally free $\alpha$-twisted sheaves of rank one or, equivalently, $\mathcal{A}$-modules of rank $d(\mathcal{A})=\sqrt{\textrm{rk}(\mathcal{A})}$ those can be ignored. Indeed, as in the untwisted case, line bundles on integral subvarieties are stable with respect to any choice of a polarisation and any of the notions of stability. Also, eventually, only the birational type is relevant for the splitting of Brauer classes.

Alternatively, the twisted Picard schemes can be constructed following the classical arguments for the untwisted Picard schemes, cf. [6] and Remark 3.4.

These moduli spaces, e.g. of $\Lambda$-modules, are projective once the Hilbert polynomial is chosen. The Hilbert polynomial *P* that we would need to single out $\textrm{Pic}^d_\alpha (\mathcal {C}/U)$ is $P(\ell )=d(\mathcal{A})\cdot (\ell \cdot (C.h)+d+1-g(C))$. Then for a closed point $[F]\in \textrm{Pic}^d_\alpha (\mathcal {C}/U)$ corresponding to an $\mathcal{A}|_C$-module *F* on a smooth curve $[C]\in U$ we have $\chi (F\otimes \mathcal{O}(\ell h))=P(\ell )$ or, equivalently, $\textrm{ch}(F)=d(\mathcal{A}) \cdot (1+ d\cdot [\text {pt}])$ for the Chern character of *F* on *C*. Indeed, for an $\mathcal{A}|_C$-module *F* on a smooth curve $C\in U$ with $\textrm{ch}(F)=d(\mathcal{A})\cdot (1+d\cdot [pt])$, the Riemann–Roch formula gives $\chi (X,F\otimes \mathcal{O}(\ell h))=P(\ell )$.

#### Remark 3.1

On a smooth projective variety, one could alternatively work with twisted Chern characters on *X* which one would then fix as

$$\begin{aligned}\textrm{ch}_\mathcal{A}(E)=[C]+d\in (\textrm{CH}^{n-1}(X)\oplus \textrm{CH}^{n}(X))/_{\sim _{\text {num}}}\otimes \mathbb {Q}.\end{aligned}$$

For $k=\mathbb {C}$ one can just work with singular cohomology and fix $\textrm{ch}_\mathcal{A}(E)$ as a class in the cohomology $H^{2n-2}(X,\mathbb {Q})\oplus H^{2n}(X,\mathbb {Q})$. Recall from [28] that the twisted Chern character $\textrm{ch}_\mathcal{A}(E)$ is defined as $\sqrt{\textrm{ch}(\mathcal{A})}^{-1}\cdot \textrm{ch}(E)$, which for sheaves concentrated on curves is just $d(\mathcal{A})^{-1}\cdot \textrm{ch}(E)$.

Note that every *F* parametrised by $\textrm{Pic}^d_\alpha (\mathcal {C}/U)$ is a locally free $\mathcal{A}$-module on some smooth complete intersection curve *C* with $\textrm{rk}(F)=d(\mathcal{A})$. In particular, $\mathcal{A}|_C\simeq \mathcal {E}\hspace{-1.66656pt}\mathcal {n}\mathcal {d}(F)$, cf. Remark 4.1. In the language of twisted sheaves, *F* would correspond to a line bundle that is twisted with respect to the restriction of a cocycle representing $\alpha$.

Assume that *d* can be chosen such that $\textrm{Pic}^d_\alpha (\mathcal {C}/U)$ is a fine moduli space, i.e. that there exists a universal sheaf $\mathcal{P}$ on $\mathcal {C}\times _U\textrm{Pic}^d_\alpha (\mathcal {C}/U)$. By definition, $\mathcal{P}$ is a module over the pull-back $\mathcal{A}'$ of $\mathcal{A}$ via the projection3.2The class $\alpha '\in \textrm{SBr}(\mathcal {C}\times _U\textrm{Pic}^d_\alpha (\mathcal {C}/U))$ of $\mathcal{A}'$ is then the pull-back of $\alpha$, i.e. its image under^2^We will use the same notation for the corresponding classes in the Brauer groups ${\textrm{Br}}(X)$, ${\textrm{Br}}(\mathcal {C})$, and ${\textrm{Br}}(\textrm{Pic}^d_\alpha (\mathcal {C}/U)\times _U\mathcal {C})$.

Note that the restriction of $\mathcal{P}$ to $[F]\times C\subset \textrm{Pic}^d_\alpha (\mathcal {C}/U)\times _U\mathcal {C}$ is the sheaf *F* on *C*. Thus, $\mathcal{P}$ is a locally free $\mathcal{A}'$-module of rank $d(\mathcal{A}')$ and, therefore, the natural map  is an isomorphism. Hence, the image $\alpha '=[\mathcal{A}']\in {\textrm{Br}}(\textrm{Pic}^d_\alpha (\mathcal {C}/U) \times _U\mathcal {C})$ of $\alpha \in {\textrm{Br}}(X)$ is trivial.

In other words, the pull-back $\alpha _\mathcal {C}\in {\textrm{Br}}(\mathcal {C})$ is split by  which is a torsor for the abelian scheme . Equivalently, if $\eta _\mathcal {C}\in \mathcal {C}$ and $\eta _X\in X$ denote the generic points of $\mathcal {C}$ and *X*, and so in particular $\eta _\mathcal {C}$ maps to $\eta _X$ under the projection , the compositionsends $\alpha$ to the trivial class. Here, $B_\alpha ^d$ is the generic fibre of  which can also be seen as the base change of the generic fibre of  to $\eta _\mathcal {C}$. Thus, (3.1) is complemented byNote that the field extension $K(X)\subset k(\eta _C)$ is independent of $\alpha$.

### Reducing the family of curves and proof of Theorem 1.1

Even under the assumption that there exists a universal $\mathcal{A}'$-module $\mathcal{P}$, our discussion does not yet prove Theorem 1.1, because $B_\alpha ^d$ is a variety over the function field $k(\eta _\mathcal {C})$ of $\mathcal {C}$ and not over *K*(*X*). This will be addressed now.

The very ample linear system |*h*| embeds *X* into a projective space $\mathbb {P}^N$. We pick a generic linear subspace $P:=\mathbb {P}^{N-n}\subset \mathbb {P}^N$, where as before $n=\dim (X)$, and project from $P\subset \mathbb {P}^{N}$ onto a generic $\mathbb {P}^{n-1}$. This defines a rational map , which is resolved by the blow-up in $P\subset \mathbb {P}^{N}$ to a morphism . Restricting to *X*, we obtain a morphism3.3from the blow-up of *X* in the points of intersection $\{x_1,\ldots ,x_d\}=X\cap P$, where $d=\deg (X)$. The fibre of (3.3) over a point $t\in \mathbb {P}^{n-1}$ is the complete intersection curve $X\cap \overline{Pt}$. Restricting to those points with smooth fibres leads to3.4In more invariant terms, the situation can be described by $P=\mathbb {P}(W)\subset \mathbb {P}^N=\mathbb {P}(V)$ and $\mathbb {P}^{n-1}=\mathbb {P}(W_0)$, with $V=H^0(X,\mathcal{O}(h))^*$ of dimension $N+1$, a generic linear subspace $W\subset V$ of dimension $N-n+1$, and a subspace $W_0\subset V$ of dimension *n* for which the projection  is an isomorphism. Mapping a point $t=[v]\in \mathbb {P}(W_0)$ to the subspace of all sections $s\in V^*=H^0(X,\mathcal{O}(h))$ vanishing on the subspace spanned by *W* and $v\in V$ defines an embeddingWe can think of the family  as the pull-back of .

What has been achieved by this construction is that the family (3.4) of curves in *X* dominates *X* birationally, i.e. there exists exactly one curve $\mathcal {C}_t$ passing through the generic point of *X*. In particular, the function fields of $\mathcal {C}_0$ and *X* can be identified: $K(\mathcal {C}_0)=k(\eta _{\mathcal {C}_0})=k(\eta _X)=K(X)$. Another feature of the construction is that the projection  admits a section. Indeed, each of the exceptional divisors $E_i\subset \textrm{Bl}_{\{x_i\}}(X)$ over the point $x_i\in X$ defines one.

At this point we change our *X* and replace it by its blow-up in one of the points, say $x_1\in X$. Nothing in the above changes except that now the new *X* contains a divisor $Y\subset X_{\text {sm}}\subset X$ isomorphic to $\mathbb {P}^{n-1}$, namely the old $E_1$, which also functions as a section of .

#### Remark 3.2

The idea to use families of complete intersection curves to study Brauer classes is not new. For example, Colliot-Thélène [14, Lem. 1] proves the existence of (3.4) and Lieblich [34, Lem. 4.2.1.1] shows how to relax the assumption on the ground field.

The restriction of (3.2) to $\mathcal {C}_0$, which is nothing but3.5can now be viewed as a torsor for the abelian scheme3.6Since the projection  is a birational morphism, the generic fibres of (3.5) and of (3.6), which we denote by $B_\alpha$ resp. *A*, can both be viewed as varieties over $k(\eta _X)=K(X)$.

The rest of the argument is as before. The restriction $\mathcal{P}_0$ of the universal sheaf $\mathcal{P}$ on $\textrm{Pic}^d_\alpha (\mathcal {C}/U)\times _U\mathcal {C}$ to the subscheme $\textrm{Pic}^d_\alpha (\mathcal {C}_0/U_0)\times _{U_0}\mathcal {C}_0\subset \textrm{Pic}^d_\alpha (\mathcal {C}/U)\times _U\mathcal {C}$ is locally free of rank $d(\mathcal{A})$ and a module over the pull-back of the Azumaya algebra representing the Brauer class $\alpha$ under (3.5).

It remains to address the existence of a universal sheaf. This is the following result.

#### Lemma 3.3

There exists a universal bundle over $\textrm{Pic}^d_\alpha (\mathcal {C}_0/U_0)\times _{U_0}\mathcal {C}_0$.

#### Proof

We use the standard criterion for the existence of universal families of stable sheaves, cf. [26, Ch. 4.6]: For this we view $\textrm{Pic}_\alpha ^d(\mathcal {C}_0/U_0)$ as a sub-scheme of the moduli space of twisted sheaves with the same numerical invariants as the sheaves parametrised by $\textrm{Pic}^d_\alpha (\mathcal {C}_0/U_0)$ viewed as sheaves on *X*. Then a universal sheaf $\mathcal{P}$ on $\textrm{Pic}_\alpha ^d(\mathcal {C}_0/U_0)\times _{U_0}\mathcal {C}_0$, twisted with respect to the pull-back of $\alpha$, exists if one finds a formal linear combination $u=\sum a_i[G^i]$ of $\alpha ^{-1}$-twisted sheaves on $X_{\textrm{sm}}$ (or a class in the corresponding Grothendieck group) with $\chi (F\otimes u)=\sum a_i\cdot \chi (F\otimes G^i)=1$ for $F\in \textrm{Pic}^d_\alpha (\mathcal {C}/U)$.

Now we choose the hypersurface $\mathbb {P}^{n-1}\simeq Y\subset X_{\text {sm}}\subset X$ introduced above. Then $\alpha |_Y=1$ and hence $\mathcal{O}_Y$ can be considered as an $\alpha ^{-1}|_Y$-twisted line bundles. (Using Azumaya algebras, this can also be expressed by saying that $\mathcal{A}|_Y\simeq \mathcal {E}\hspace{-1.66656pt}\mathcal {n}\mathcal {d}(E)$ for some locally free sheaf *E*.) Since $Y\subset X$ is contained in the smooth locus of *X*, there exists a finite locally free resolution  of $i_*\mathcal{O}_Y$ by locally free $\alpha$-twisted sheaves $G^i$, cf. [11]. Then set $u=\sum (-1)^i[G^i]$ and compute

$$\begin{aligned}\chi (F\otimes u)=\chi (F\otimes i_*\mathcal{O}_Y)=(\mathcal {C}_t.Y)=1,\end{aligned}$$

as the pre-image of *Y* under  is a section of . $\square$

This concludes the proof of Theorem 1.1.

#### Remark 3.4

We have chosen to use the general existence criterion for universal sheaves on moduli spaces of stable sheaves, cf. [26, Ch. 4.6], as it has the advantage that similar arguments can be applied to produce varieties splitting Brauer classes by means of moduli spaces of bundles on curves.

The existence of the twisted Poincaré bundle can also, and maybe more easily, deduced from arguments following the classical theory of Picard schemes, cf. [6]: The relative twisted Picard scheme  admits a universal $(1,\alpha )$-twisted family $\mathcal{P}$ on $\textrm{Pic}_\alpha (\mathcal {C}/U)\times _U\mathcal {C}$ if the family  has a section  with $\alpha |_{s(U)}=1$ in $H^2(s(U),{{\mathbb {G}}}_m)$. Alternatively, arguments of Simpson [43, Thm. 4.7] could be exploited.

### Comments on the assumptions

Our construction is geometric and we need our ground field to be algebraically closed. For example, even in the untwisted version, a Poincaré line bundle may not exist if *k* is not algebraically closed. More geometrically, if *X* is a smooth projective curve over a field which is not algebraically closed, then ${\textrm{Br}}(X)$ might be non-trivial but the construction of system of curves does not make sense.

As our arguments use the existence of the (twisted) Picard variety of curves contained in the variety *X*, it seems unlikely that the ideas could be extended to also cover ramified classes in ${\textrm{Br}}(K(X))$, i.e. those that are not contained in ${\textrm{Br}}(X)$.

#### Remark 3.5

As a continuation of the discussion in Sect. 2.3 and especially Remark 2.5, we note that the techniques above can be modified to prove similar results for varieties *X* with a dominating family of curves , potentially of much lower genus than the complete intersection curves used above. Analogously to Proposition 2.4, one would then prove splitting of Brauer classes over torsors for abelian varieties *A* over some finite extension $K'/K(X)$ of the function field, but again with a certain condition on the period of the Brauer class.

## Period–index problem

This section contains the proof of Theorem 1.2.

### Bounding the index of central simple algebras

The main argument to prove Theorem 1.2 can be given using twisted sheaves, sheaves on Brauer–Severi varieties, or sheaves on gerbes. We chose to present it in the language of Azumaya algebras, as it makes the argument most transparent. We begin by recalling the following basic fact.

Assume *A* is an Azumaya algebra over a field *K* and *V* is a module over *A*. Then

$$\begin{aligned}(\textrm{ind}(A)\cdot d(A))\mid \dim _K(V).\end{aligned}$$

Indeed, writing $A\simeq M_\ell (D)$ and using Morita equivalence, we know that *V* is of the form $W^{\oplus \ell }$ for some module *W* over the division algebra *D*. Since any *D*-module is free, this proves $V\simeq D^{\oplus \ell \cdot m}$ for some *m* and, therefore, $\textrm{ind}(A)\cdot d(A)=\dim _K(D)^{1/2}\cdot \dim _K(D)^{1/2}\cdot \ell$ divides $\dim _K(V)=\dim _K(D)\cdot (\ell \cdot m)$.

So, in order to prove Theorem 1.2, it suffices to find for each Azumaya algebra $\mathcal{A}$ on *X* an *A*-module $V_\mathcal{A}$ with $\dim _K(V_\mathcal{A})=d(A)\cdot \textrm{per}(A)^e$ for a certain fixed *e*. Here, $A=\mathcal{A}_{K}$ denotes the Azumaya algebra over the function field $K=K(X)$ obtained as the generic fibre of $\mathcal{A}$.

The basic idea is to produce such an *A*-module *V*, cf. (4.3), as the space of global sections

4.1 $$\begin{aligned} H^0(\textrm{Pic}_\alpha ^d(\mathcal {C}_0/U_0)_K,\mathcal{P}|_{\textrm{Pic}_\alpha ^d(\mathcal {C}_0/U_0)\times {\eta _{\mathcal {C}_0}}}\otimes M) \end{aligned}$$

of the restriction of the universal sheaf $\mathcal{P}$ twisted by an appropriate line bundle *M* and to control its dimension. So, ultimately *V* is a space of theta functions on a twisted Picard variety. Since $\mathcal{P}$ is a sheaf of modules over the pull-back of $\mathcal{A}$, its space of global sections is indeed an *A*-module.

#### Remark 4.1

The above can also be phrased in more geometric terms. Assume *E* is a locally free sheaf on *X* and a module over the Azumaya algebra $\mathcal{A}$. If $\textrm{rk}(E)=d(\mathcal{A})$, then the natural injection  is generically an isomorphism. Moreover, since both sheaves have trivial determinant, it is an isomorphism in codimension one and hence, at least when *X* is smooth, everywhere. In particular, the class of $\mathcal{A}$ is trivial. The referee pointed out that one could also argue purely algebraically, using the double centralizer theorem, see e.g. [22, II. Thm. 4.3], on affine open sets.

The basic idea to construct modules over Azumaya algebras (or twisted sheaves) of small rank to bound the index has been exploited before by Lieblich [34, Prop. 4.2.1.3]. Instead of trying to find rational points of moduli spaces to produce those, we propose here to use spaces of global sections of certain twisted sheaves.

### Direct image of the twisted Poincaré bundle and proof of Theorem 1.2

With the notation of the last section, we consider a birationally dominating family of very ample complete intersection curvesparametrised by an open subset $U_0\subset \mathbb {P}^{n-1}$.

According to Lemma 3.3, we can assume that there exists a universal $(1,\alpha )$-twisted sheaf $\mathcal{P}$ on $\textrm{Pic}^d_\alpha (\mathcal {C}_0/U_0)\times _{U_0}\mathcal {C}_0$ over $U_0$. If $\textrm{Pic}^d_\alpha (\mathcal {C}_0/U_0)$ is viewed as a sub-scheme of a moduli space of sheaves over the Azumaya algebra $\mathcal{A}$ picked to represent the class $\alpha \in \textrm{SBr}(X)$, then the universal sheaf $\mathcal{P}$ is a module over the pull-back of $\mathcal{A}$ under the projection . As always, the universal sheaf is only unique up to twist coming from $\textrm{Pic}^d_\alpha (\mathcal {C}_0/U_0)$ and the first step consists of twisting $\mathcal{P}$ appropriately so that it parametrises (the analogue of) degree zero line bundles on $\textrm{Pic}_\alpha ^d(\mathcal {C}_0/U_0)$.

To simplify notation, let us restrict the situation to the generic point $\xi \in U_0$. We denote the generic fibre of $\mathcal {C}_0$ by *C*, a curve over $K_0:=k(\xi )=K(U_0)$, and set

$$\begin{aligned}P:=\textrm{Pic}_\alpha ^d(\mathcal {C}_0/U_0)_{\xi }=\textrm{Pic}_{\alpha |_C}^d(C),\end{aligned}$$

which is a torsor for the abelian variety $\textrm{Pic}^0(C)$ over $K_0$.

Intersecting *C* with one of the exceptional divisors $E_i\subset \textrm{Bl}_{\{x_i\}}(X)$ produces a closed point $x\in C$, which can also be viewed as the generic point of $E_i$. As $E_i\cap \mathcal {C}_0$ is a section of , the residue field of *x* is $k(x)=K_0$. Moreover, the pull-back $A_x$ of $\mathcal{A}$, which is the pull-back of $\mathcal{A}\otimes k(x_i)$ under the projection , is Morita trivial, as $k(x_i)\simeq k$ is algebraically closed by assumption. Hence, $A_x\simeq M_{d(A)}(K_0)$.

The restriction $\mathcal{P}|_{P\times \{x\}}$ of the universal sheaf can then be seen as a a locally free sheaf of modules on $P\simeq P\times \{x\}$ over the Azumaya algebra $\mathcal{O}_P\boxtimes A_x\simeq M_{d(A)}(\mathcal{O}_{P\times \{x\}})$ and is thus of the form $L^{\oplus d(A)}$ for some invertible sheaf *L* on $P\times \{x\}$. Thus, tensoring the twisted sheaf $\mathcal{P}$ on $P\times C$ by the invertible sheaf $L^*\boxtimes \mathcal{O}$ results in a universal sheaf $\mathcal{P}'$ on $P\times C$ for which the restriction to $P\times \{x\}$ is $\mathcal{O}^{\oplus d(A)}$.

If *M* is any ample invertible sheaf on *P*, then for $i>0$

$$\begin{aligned}H^i(P\times \{x\},(\mathcal{P}'\otimes (M\boxtimes \mathcal{O}))|_{P\times \{x\}})=H^i(P\times \{x\},(M\boxtimes \mathcal{O})^{\oplus d(A)}|_{P\times \{x\}})=0,\end{aligned}$$

which by semi-continuity implies the same vanishing for the restriction to $P\times \{\eta \}$, where $\eta$ is the generic point of *C*, whose residue field is the function field $K=K(X)$.

The next step is to find an appropriate *M* for which $\chi (P\!\times \!\{\eta \}, (\mathcal{P}'\!\otimes \! (M\boxtimes \mathcal{O}))|_{P\!\times \!\{\eta \}})$ can be bounded. For this we consider the natural morphism4.2given by tensor product (over $\mathcal{A}|_C$) , where $D=\textrm{per}(\alpha )\cdot d$. As $\alpha ^{\textrm{per}(\alpha )}=1$, the twisted Picard variety on the right hand side is actually untwisted, i.e. isomorphic $\textrm{Pic}^a(C)$ for a certain *a*. Since $C(K_0)\ne \emptyset$, the latter is isomorphic to $\textrm{Pic}^0(C)$ and admits an ample invertible sheaf $\mathcal{O}(\Theta )$ satisfying $(\Theta )^{g(C)}=g!$, i.e. an invertible sheaf inducing a principal polarisation.

Thus, (4.2) induces a finite surjective morphismof degree $\textrm{per}(\alpha )^{2g(C)}$ and the pull-back $M:=\varphi ^*\mathcal{O}(\Theta )$ is ample with top intersection number $(M)^{g(C)}= (\Theta )^{g(C)}\cdot \deg (\varphi )=g!\cdot \textrm{per}(\alpha )^{2g(C)}$. For this *M* we then have by Riemann–Roch and standard vanishing results for abelian varieties

$$\begin{aligned}  &   \dim _{K} H^0(P\times \{\eta \},(\mathcal{P}'\otimes (M\boxtimes \mathcal{O}))|_{P\times \{\eta \}}) =h^0(P\times \{\eta \},(\mathcal{P}'\otimes (M\boxtimes \mathcal{O}))|_{P\times \{\eta \}})\\  &   \quad =\chi (P\times \{\eta \},(\mathcal{P}'\otimes (M\boxtimes \mathcal{O}))|_{P\times \{\eta \}}) =\chi (P\times \{x\},(\mathcal{P}'\otimes (M\boxtimes \mathcal{O}))|_{P\times \{x\}})\\  &   \quad =\chi (P\times \{x\},(M\boxtimes \mathcal{O})^{\oplus d(A)}|_{P\times \{x\}}) =d(A)\cdot \textrm{per}(\alpha )^{2g(C)}. \end{aligned}$$

Thus, the *A*-module

4.3 $$\begin{aligned} V:=H^0(P\times \{\eta \},(\mathcal{P}'\otimes (M\boxtimes \mathcal{O}))|_{P\times \{\eta \}}) \end{aligned}$$

is of dimension

$$\begin{aligned} \dim _K(V)= d(A)\cdot \textrm{per}(\alpha )^{2g(C)}, \end{aligned}$$

which by the discussion in Sect. 4.1 implies

4.4 $$\begin{aligned} \textrm{ind}(A)\mid \textrm{per}(\alpha )^{2g(C)}. \end{aligned}$$

Hence, this concludes the proof of Theorem 1.2.

## Data Availability

No new data were created or analysed in this study. Data sharing is not applicable to this article.

## Footnotes

1 It is enough that $(C.f)=k\cdot m$ with also *k* coprime to $|\alpha |$, which is slightly easier to arrange.

2 The special Brauer group can be avoided here. Instead of a Brauer class, one simply fixes a $\mu _n$-gerbe representing it. The special Brauer group only keeps track of all possible choices of $\mu _n$-gerbes.
